# Incomplete Trifascicular Block and Mobitz Type II Atrioventricular Block in COVID-19

**DOI:** 10.7759/cureus.10461

**Published:** 2020-09-15

**Authors:** James C Gubitosa, Phoenix Xu, Ahmed Ahmed, Kathleen Pergament

**Affiliations:** 1 Internal Medicine, University Hospital - Rutgers New Jersey Medical School, Newark, USA

**Keywords:** covid 19, clinical cardiology, clinical cardiac electrophysiology, electrocardiogram (ecg/ekg), covid-19 pneumonia, trifascicular block, mobitz type 2 av block, bifascicular block, qtc prolongation, second-degree heart block

## Abstract

A 74-year-old female with a history of diabetes presented with chest pain and shortness of breath for two days. She was hypoxic to an oxygen saturation of 60% in the emergency department, requiring bilevel positive airway pressure (BiPAP) to maintain saturations. Chest X-ray demonstrated bilateral hazy opacities suspicious for viral pneumonia. Coronavirus disease 2019 (COVID-19) was confirmed. Right bundle branch block (RBBB) with left anterior fascicular block was noted on admission electrocardiogram (ECG). Cardiac enzymes and brain natriuretic peptide levels were within normal limits. After noting frequent pauses on telemetry, a repeat ECG was performed that demonstrated RBBB with left posterior fascicular block as well as second-degree atrioventricular block (Mobitz type II). Transcutaneous pacing pads were placed, and atropine was placed at the bedside. Cardiac enzymes remained negative. Interleukin-6 levels were elevated at 159 pg/mL. Hydroxychloroquine was deferred due to the patient’s arrhythmia and prolonged QTc. Tocilizumab was deferred due to the patient’s age. The patient’s oxygen requirements and mental status continued to worsen. She continued to desaturate despite maximal BiPAP therapy and eventually died.

Cardiac involvement in COVID-19, whether caused primarily by the virus, secondary to its clinical sequelae, or even due to its treatment, cannot be ignored. Further high-quality research is needed to clarify the cardiac pathophysiology. Thorough cardiac exams with electrocardiographic correlation should be performed on all patients with COVID-19. Clinicians should not hesitate to consult cardiovascular services in the event of abnormality.

## Introduction

Coronavirus disease 2019 (COVID-19), caused by severe acute respiratory syndrome coronavirus 2 (SARS-CoV-2), has affected people worldwide, with the United States having the most reported cases thus far [1]. As with previous iterations of coronavirus (SARS-CoV, Middle Eastern respiratory syndrome [MERS-CoV], and seasonal coronavirus), the respiratory system has been noted to be the primary target. In data from Wuhan, China, 19.7% of hospitalized COVID-19 patients experienced acute respiratory distress syndrome (ARDS); however, cardiac abnormalities due to COVID-19 were also found to be prevalent. Although the COVID-19 literature is preliminary, case reports have described myocarditis as an observed complication [2,3]. Moreover, in one study, 16.7% of patients experienced an arrhythmia and 7.2% experienced cardiac injury, while other studies demonstrated 23% of COVID-19 patients developed heart failure [4,5]. A recent meta-analysis showed MERS-CoV and SARS-CoV did not cause this level of cardiac involvement, while clinical cardiac manifestations of COVID 19 are becoming apparent as the disease continues to affect patients [6]. Possible mechanisms of COVID-19-induced cardiac complications include hypoxia-induced myocardial injury, direct viral invasion, and inflammatory processes. We present the case of a patient with COVID-19 found to have profound conducting system disease resulting in a rare constellation of electrocardiographic abnormalities, namely incomplete trifascicular block and Mobitz type II atrioventricular (AV) block [7]. 

## Case presentation

A 74-year-old woman with a history of diabetes presented with fever, chest pain, and shortness of breath for two days. She described the chest pain as a dull, intermittent, and non-radiating ache. Her shortness of breath also worsened with exertion. Prior to presentation, she was able to freely walk several blocks but was now limited to short distances of four to eight feet uninhibited. On admission, she was hypoxic, with an oxygen saturation via pulse oximetry of 60% on room air. Oxygen saturation initially improved with maximum oxygen settings via nasal cannula, non-rebreather mask, and prone positioning. The patient’s respiratory status, however, soon worsened, and she required bilevel positive airway pressure (BiPAP). On exam, she exhibited normal heart sounds without any lower extremity edema, jugular venous distention (JVD), or other signs of decompensated cardiac function. She also denied tobacco use, alcohol use, substance use, any personal cardiac history, or family history of cardiac disease. The patient also reported that her son and husband were being treated for COVID-19 at an outside hospital. Her only home medication was metformin 1,000 mg twice daily.

**Figure 1:**
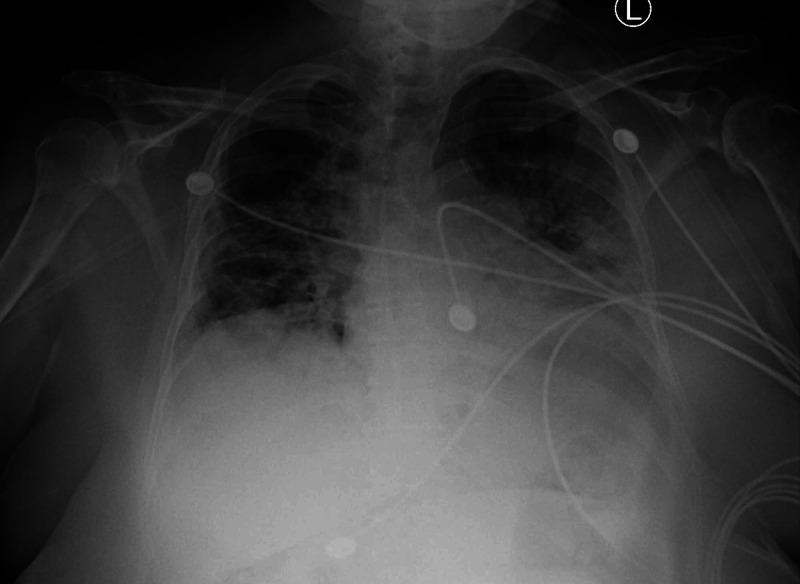
Anteroposterior (AP) X-ray of the chest upon admission X-ray demonstrating bilateral hazy opacities suspicious for a viral pneumonia

Labs were significant for a D-dimer of 1,471 ng/mL (reference: 90-500 ng/mL), lactate dehydrogenase (LDH) of 635 U/L (reference: 120-250 U/L), leukocyte count of 12 x 10^3^/µL (reference: 4-11 x 10^3^/µL) with lymphopenia, and ferritin of 618 ng/mL (reference: 30-150 ng/mL). Her chest X-ray was notable for bilateral ground-glass opacities suspicious for viral pneumonia (Figure 1). No further chest imaging was performed.

**Figure 2:**
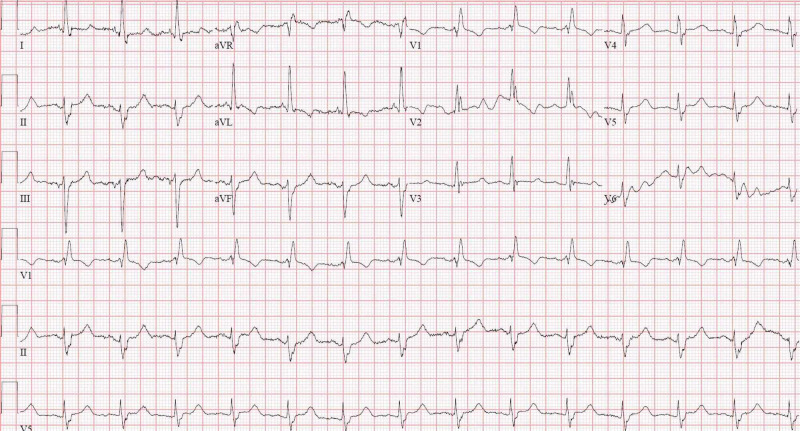
Twelve-lead electrocardiogram (ECG) upon admission ECG demonstrating bifascicular block (composed of RBBB and LAFB) as well as prolonged QTc RBBB, right bundle branch block; LAFB, left anterior fascicular block

Her admission electrocardiogram (ECG) showed bifascicular block consisting of a right bundle branch block (RBBB) and left anterior fascicular block (LAFB) as well as a prolonged QTc (522 milliseconds, calculated via the Framingham formula) (Figure 2). 

**Figure 3:**
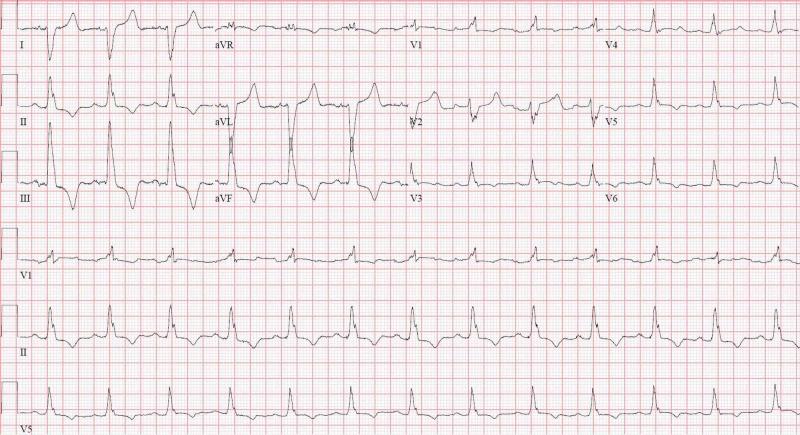
Twelve-lead electrocardiogram (ECG) taken shortly after admission ECG demonstrating altered bifascicular block (RBBB with LPFB) and prolonged QTc RBBB, right bundle branch block; LPFB, left posterior fascicular block

**Figure 4:**
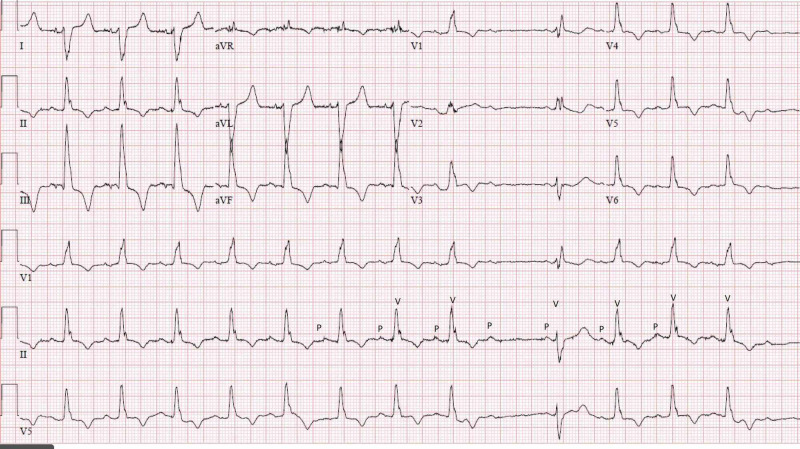
Twelve-lead electrocardiogram (ECG) taken several hours after admission ECG demonstrating bifascicular block (RBBB with LPFB, second-degree HB (Mobitz type II), and prolonged QTc. "P" in lead II identifies observed P-waves, while "V" identifies observed QRS complexes. There is an observed dropped QRS after the fourth noted "P", without progressive PR lengthening RBBB, right bundle branch block; LPFB, left posterior fascicular block; HB heart block

There was no baseline ECG present to compare. Given high suspicion, she was tested for COVID-19 and found to be positive via real-time polymerase chain reaction (RT-PCR). During her hospitalization, the telemetry monitor repeatedly showed irregularity and a repeat ECG showed an altered bifascicular block consisting of RBBB and left posterior fascicular block (LPFB) (Figure 3) as well as episodes of Mobitz type II second-degree AV block (Figure 4).

Cardiac enzymes and her brain natriuretic peptide (BNP) level were followed and found to be normal. Interleukin-6 (IL-6) levels were checked and noted to be elevated at 159.3 pg/mL (reference: 0.0-12.2 pg/mL). The differential diagnosis at the time included early and/or localized ischemia, generalized hypoxia, and direct conducting system damage (such as in myocarditis or other inflammatory processes). In addition to her hypoxic respiratory failure secondary to COVID-19 pneumonia, given the evidence of disease in all three fascicles, a diagnosis of incomplete trifascicular block was made. Therapeutic enoxaparin (1.5 mg/kg daily) was empirically initiated for suspected pulmonary embolism given her elevated D-dimer and persistent hypoxia in the setting of COVID-19. At the time, hydroxychloroquine (HCQ) was a recommended therapy; however, she was not a candidate due to her prolonged QTc. Moreover, she was not a candidate for tocilizumab due to her advanced age. Remdesivir was not yet seeing widespread use and thus was not considered. Her mental status and hypoxia continued to worsen during her hospital course, requiring maximal BiPAP therapy. Because of facility-based restrictions at the time, echocardiography to assess for regional wall motion abnormalities was unable to be performed. Telemetry monitoring demonstrated worsening bradycardia and resultant hypotension. As her status worsened, the patient declined both temporary venous pacing and intubation. She later experienced pulseless electrical activity (PEA) arrest and was pronounced dead. 

## Discussion

Our patient exhibited both newfound and extensive conducting system disease. No personal or familial history of cardiac disease was known, and there were no prior electrocardiograms available to compare. The criteria of RBBB were met via visualization of a QRS > 120 ms, the presence of an rSR’ pattern in V1-V3, an upright QRS in V1, and a slurred S-wave in V5-6. LAFB was established given left axis deviation in the setting of a lack of left ventricular hypertrophy (LVH). LPFB was established via later right axis deviation. The criteria of bifascicular block were met in Figure 2, which demonstrated an RBBB plus LAFB, as well as in Figure 3, which demonstrated a RBBB plus LPFB. The diagnosis of incomplete trifascicular block was established given the evidence of disease in all three fascicles (right, left anterior, and left posterior). Lastly, the diagnosis of second-degree AV block (Mobitz type II) was made given frequent dropped QRS complexes seen in Figure 4 (as well as telemetry monitoring) without evidence of progressive PR-interval prolongation, suggesting infra-Hisian disease.

Arrhythmia in the setting of coronary artery disease (CAD) is seen frequently. The right bundle branch is perfused predominantly by the septal branch of the left anterior descending artery (LAD). Both fascicles of the left bundle branch are perfused by the LAD or AV-nodal artery (which arises from the right coronary artery or RCA in 80% of patients). The His bundle is predominantly supplied by the RCA, but also by the LAD septal branches in 10% of patients [8,9]. Triple-fascicle disease and complete heart block have been reported in cases of acute myocardial infarction (MI). This finding can present with anterior MI, suggesting LAD and septal perfusing branch pathology, but has also been seen in inferior MI, suggesting RCA and AV-nodal artery compromise [10]. Mobitz type II AV blocks most commonly present within or below the His bundle [8,9]. The abnormalities highlighted in this patient’s ECGs demonstrate an overall picture of conducting system disease below the level of the AV node.

The patient was an elderly female with a known history of diabetes, both of which are risk factors for cardiovascular disease. It is possible that such risk factors contributed to her overall cardiovascular health; however, her sudden decline with no previous cardiac history and worsening irregularity on telemetry during hospitalization make a complication secondary to COVID-19 more likely than mere coincidence. Furthermore, an acute ischemic event in our patient was unlikely given the lack of cardiac enzyme elevation. It is possible our patient had undiagnosed existing CAD. Her 10-year atherosclerotic cardiovascular disease (ASCVD) risk was calculated to be 29.1% at the time. Increased sympathetic tone present in acute illness can lead to increased myocardial oxygen demand which, coupled with the patient’s persistent hypoxia due to COVID-19-associated pneumonia and potential ASCVD may have contributed to or exacerbated her arrhythmia.

Myocarditis has been suspected in several cases of COVID-19 [2,3,11]. In rabbits, rabbit coronavirus (RbCV) was shown to be associated with a rapidly progressive myocarditis. Samples of cardiac myocytes demonstrated necrosis and mononuclear infiltrates but no visible viral inclusion bodies. However, RbCV was able to be isolated from the samples [10]. In a study of pathological findings in deceased COVID-19 patients, myocyte samples again demonstrated mononuclear infiltrates without viral inclusion bodies [11]. Increased circulating levels of IL-6, which occur due to the “cytokine storm” seen in COVID-19, may cause immune-mediated damage to cardiac myocytes. While our patient had elevated IL-6 levels, there was again no evidence of myocardial damage. Moreover, her troponins and BNP level remained normal during her hospital course. She did have symptoms of chest pain prior to hospitalization; however, it did not recur while in the hospital and her dyspnea was attributed to the COVID-19-associated pneumonia.

The management of complex conducting system disease is best presented by breaking down the individual identified disorders that presented in our patient. Per the 2018 American College of Cardiology (ACC), American Heart Association (AHA), and Heart Rhythm Society (HRS) guidelines, ambulatory electrocardiographic monitoring would be recommended (class IIb) for asymptomatic patients with extensive conducting system disease such as bifascicular block or trifascicular block. However, patients with documented second-degree Mobitz type II heart block are recommended (class I) to receive permanent cardiac pacing [12]. Given the presence of both of these disorders in our patient, permanent cardiac pacing would have taken precedence as the therapy of choice.

## Conclusions

The etiology of the advanced conducting system disease seen in our patient is likely multifactorial. The causative factors were likely hypoxia and increased sympathetic tone (which can worsen AV conduction in the setting of second-degree, type II AV block) in the setting of critical illness due to COVID-19. This may have been exacerbated by the unknown, but plausible presence of pre-existing ASCVD since the patient was an elderly diabetic with an elevated ASCVD risk score. If the patient had not expired, she would have benefited from further cardiac workup including echocardiography, and possibly cardiac catheterization or electrophysiologic studies. Additionally, if the patient wanted to pursue indicated therapies, she would also have received permanent cardiac pacing prior to discharge.

Cardiac involvement in COVID-19, whether it is caused by the virus versus its clinical sequelae, cannot be overlooked. Research is needed to elucidate the pathophysiology of COVID-19-associated conducting system disease. In the interim, it is important to be aware of the increased prevalence of arrhythmia and cardiac complications in COVID-19 patients. Cardiac exams should be performed on all patients with COVID-19 to assess for altered cardiac function. Clinicians caring for patients with COVID-19 should also be mindful of abnormal electrocardiographic findings. Expedient assessment of any noted cardiac abnormalities by cardiovascular services should be encouraged. It is our hope that these measures will prevent and decrease the amount of cardiovascular morbidity and mortality in COVID-19 patients.
